# Chylous ascites as a late complication of one anastomosis gastric bypass-minigastric bypass: case report

**DOI:** 10.1186/s12893-020-00758-z

**Published:** 2020-05-06

**Authors:** Ibrahim Abu Shakra, Maxim Bez, Amitai Bickel, Walid Kassis, Samer Ganam, Fahed Merei, Nour Karra, Khatib Kamal, Doron Fischer, Eli Kakiashvili

**Affiliations:** 1 Department of Surgery A, Galilee Medical Center, Nahariya, Israel; 2 Medical Corps, Israel Defense Forces, Ramat Gan, Israel; 3 Faculty of Medicine in the Galilee, Bar-Ilan University, Safed, Israel; 4 Department of Radiology, Galilee Medical Center, Nahariya, Israel

**Keywords:** Case report, Bariatric surgery, Gastric bypass, Internal hernia, Chylous ascites

## Abstract

**Background:**

One anastomosis gastric bypass- minigastric bypass (OAGB-MGB) is an emerging bariatric surgery that is being endorsed by surgeons worldwide. Internal herniation is a rare and dreaded complication after malabsorptive bariatric procedures, which necessitates early diagnosis and intervention.

**Case presentation:**

We describe a 29-year-old male with chylous ascites caused by an internal hernia 8 months following laparoscopic one anastomosis gastric bypass. An abdominal CT showed enlargement of lymph nodes at the mesentery, with a moderate amount of liquid in the abdomen and pelvis. An emergent exploratory laparoscopic surgery demonstrated an internal hernia at the Petersen’s space with a moderate quantity of chylous ascites. The patient made an uneventful recovery after surgery.

**Conclusions:**

Internal herniation can occur after OAGB-MGB and in extremely rare cases lead to chylous ascites. To our knowledge, this is the first reported case of chylous ascites following one anastomosis gastric bypass.

## Background

One-anastomosis gastric bypass- minigastric bypass (OAGB-MGB) is an emerging bariatric surgery technique currently endorsed by surgeons worldwide [1, 2]. Similar to Roux-en-Y gastric bypass (RYGB), OAGB-MGB is both a restrictive and malabsorptive operation that involves the creation of a single anastomosis. OAGB-MGB is done by dividing the stomach between the antrum and body on the lesser curvature. This creates a pouch that is anastomosed to a small bowel loop as an antecolic and antegastric loop gastrojejunostomy. OAGB-MGB is considered a simpler and faster procedure compared with RYGB, while having similar post-operative weight loss rates [3, 4]. A recent meta-analysis reported higher excess weight loss achievable with OAGB-MGB at 5 years follow-up [5].

Early complication rates of OAGB-MGB range from 0.5 to 3.1% [3]. Early major complications requiring reoperation were reported in 1.3% of patients. Late complications, which occur in 10% of patients, include stomal stenosis, marginal ulcers, protein calorie malnutrition, bile reflux, and internal herniation of the small bowel which leads to bowel obstruction. In extremely rare cases internal hernia can cause chylous ascites following RYGB [6]. This, however, has not been observed in OAGB-MGB.

## Case presentation

We report a 29-year-old male, otherwise healthy, who presented to our emergency department, complaining of severe diffuse abdominal pain that had developed over the last 24 h, and had become progressively worse with time. The patient did not report any constipation, diarrhea, or distention despite having nausea and vomiting. He had undergone laparoscopic OAGB-MGB in a different medical center 8 months prior to his admission (Body mass index (BMI) prior to the surgery: 42 kg/m^2^; weight loss 53 kg). On physical examination, the patient’s vital signs were within normal range. The patient had no fever. Abdominal auscultation revealed normal bowel sounds. Upon abdominal palpation, the patient complained of diffuse tenderness. His lab results, including lactate, amylase and lipase were all within normal limits.

**Fig. 1:**
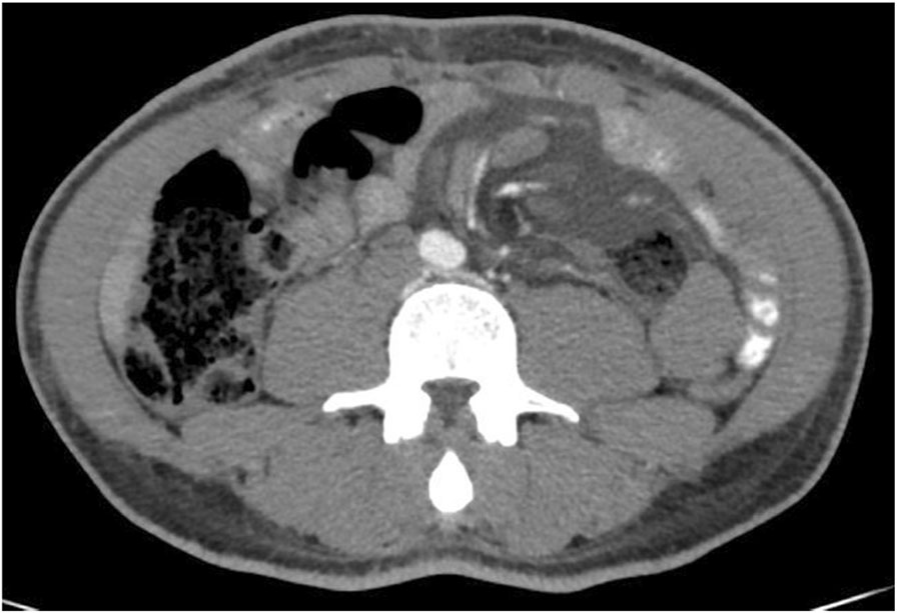
Abdominal computed tomography with intravenous contrast showing diffuse enlargement of mesenteric lymph nodes

**Fig. 2:**
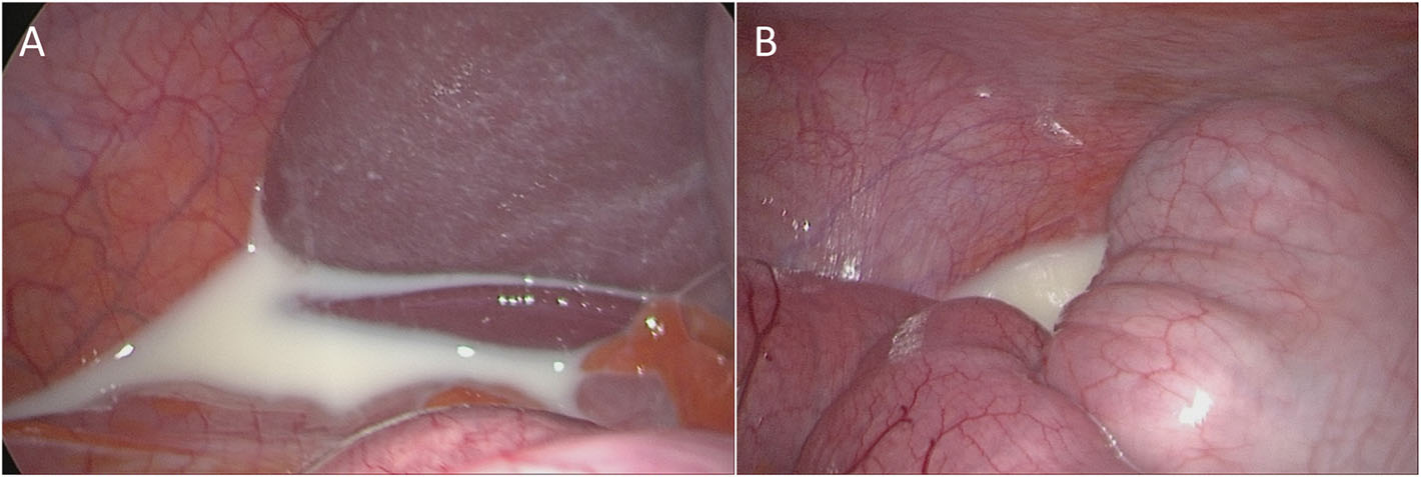
Laparoscopic aspect. Chylous ascites can be seen in the peritoneum (**a**) and in the pelvic cavity (**b**) due to a lymphatic leak

A computed tomography scan of the abdomen and the pelvis following oral and intravenous contrast administration demonstrated no evidence of intestinal obstruction or free air. Enlarged lymph nodes at the root of the mesentery were observed, with a moderate amount of free liquid of unknown origin in the abdomen and pelvis (Fig. 1). The above finding led to the decision to perform an emergent exploratory laparoscopic surgery, which demonstrated a moderate quantity of chylous ascites. Further exploration revealed the presence of an internal hernia that involved the alimentary loop, which was misplaced within the Petersen’s space with engorged lymphatic vessels within the mesenteric root (Fig. 2). Several peritoneal lavages were performed, the internal hernia was reduced and the opening was narrowed. A sample of the ascites fluid demonstrated a triglyceride level of 159 mg/dl. The postoperative course was satisfactory and the patient was discharged without symptoms at 4 days postoperative.

## Discussion and conclusions

Parallel to dramatic increases worldwide in obesity prevalence, surgical procedures aimed to induce and maintain weight loss have increased considerably over recent years. Both RYGB and OAGΒ-MGB are common, well-tolerated and effective operations for morbid obesity. Both surgeries carry similar rates of complications and reoperations, with leaks and hemorrhages being the most common due to the gastrointestinal anastomoses [5].

An internal herniation is a dangerous late complication that may occur after both RYGB and OAGB-MGB procedures (although much less commonly) and is characterized by symptoms of abdominal obstruction [7]. Clinicians need to be aware of such complication as delayed diagnosis may result in small bowel ischemia and death. The most common site for internal hernia to occur is the Petersen’s space. The incidence of an internal hernia and bowel obstruction was found to be higher following RYGB than either OAGB-MGB or sleeve gastrectomy [7]. According to a consensus statement on OAGB-MGB, 71% of the experts agreed that routine closure of the Petersen’s space should not be done routinely [8]. However, due to the increased number of reports of Petersen’s hernia after OAGB-MGB, opinions of some experts have started to shift [9].

Chylous ascites in a bariatric surgery setting generally occurs by a loop passing through the Petersen’s defect, which causes direct compression of the mesenteric vessels and fluid extravasation. In previous case reports of patients with chylous ascites following RYGB, the complication was resolved by the release of the internal hernia compressing the mesenteric lymphatic vessels [6]. Similar to these reports, the cause of chylous ascites in our patient was the direct obstruction of the lymphatic vessels, secondary to an internal hernia through the mesenteric defect at the Petersen’s space. Therefore, the release of the internal hernia causing the obstruction resulted in the resolution of the chylous ascites.

Closure of the mesenteric defect at the time of the primary surgery has been shown to reduce the occurrence of internal hernia following RYGB procedures [10]. Although the closure increases risk of early small bowel obstruction caused by kinking of the jejunojejunostomy or damage to the mesenteric vessels, this is unlikely to occur in OAGB-MGB, due to the absence of a jejunojejunostomy. Since internal herniation is a potentially serious complication, the inclusion of the routine closure of the mesenteric defect in OAGB procedures warrants evaluation.

In conclusion, internal hernia is a dangerous complication following OAGB, which in extremely rare cases can lead to chylous ascites. Surgical intervention is required to release the hernia and relieve the compression of the lymphatic vessels. Closure of the potential spaces that can lead to internal herniation should be evaluated in OAGB.

## Data Availability

Not applicable.

## Abbreviations

OAGB-MGB: One anastomosis gastric bypass- minigastric bypass
RYGB: Roux-en-Y gastric bypass
BMI: Body mass index
